# Progress in Anæsthesia

**Published:** 1888-03

**Authors:** 


### EDITORIAL.

### Progress in Anæsthesia.

Anesthesia is in some respects, at least, one of the subjects of medical science to which attaches questions that are not settled, and some that are in obscurity seem far from being cleared up.

Every thing that tends to remove difficulties, to make the way easier and more certain, should be hailed with delight by every practitioner of the healing art in any of its departments. The mitigation of pain under whatever circumstances it may occur, is greatly to be desired. But the difficulties, uncertainties, and dangers attending the use of anesthetics hitherto has been a bar to its use in many cases where it would be very desirable; the injury that may attend the use of anæsthetics is more or less impressed on the mind of every one, and very strongly with many. Any thing, either in the way of agents or administration, that will tend to lessen this liability to injury is certainly much to be desired, and is a step toward that goal to which all hope to arrive at some near point in the future. Experiment and investigation is on the alert continually to obtain the desired agent, one that will be effective and from which no injury will occur. No *new* agent of any special prominence has been introduced or even suggested recently. The mixing and compounding of the various agents that have been for a long time in use seems to have more attention; and in this direction good results seem to have been attained.

The mode of administration is a subject that ought to have had more attention than has been bestowed upon it.

A step in this direction, and a very definite one too, has recently been made by Dr. S. J. Hayes, of Pittsburgh, Pa. Dr. Hayes has been experimenting upon an apparatus for the better administration of anaesthetics for several years; and for some four or five years has been using successfully his improved apparatus; but not till the last year has he ventured or attempted to bring it before the medical profession. So fully has he become convinced of its value and importance that he has for the last few months been demonstrating its use to the physicians and dentists with whom he has come in contact; and in nearly every instance to the satisfaction and even delight of those who have witnessed its operation.

The apparatus used by Dr. Hayes is especially for the use of the liquids used for anæsthetic purposes; for this it is most complete. It regulates the percent of the vapor administered very accurately, putting it absolutely under the control of the operator, so that from one per-cent of vapor to any quantity desired, mixed with atmospheric air, may be used. From witnessing its operation in several cases recently we became strongly impressed with the superiority of the apparatus, as well as with the mode of administration, judging by the results.

The whole appliance is portable, not weighing over twelve or fifteen pounds, presenting quite a contrast to the cumbersome nitrous oxide apparatus in common use. And in the use of chloroform, or ether, or any of their mixtures, there is no odor in the room where given, which is a very great relief indeed, especially to those who are accustomed to use these agents.

We have no hesitation in saying that the apparent merit of this apparatus, and mode of administration is worthy the special attention and consideration of all who use anæsthetics.

